# Mobile Dermatology Clinics: Opportunities to Expand Medical and Surgical Care Access in Underserved Communities

**DOI:** 10.7759/cureus.102172

**Published:** 2026-01-23

**Authors:** Taha Rasul, Benjamin Cooper, Fiona S Gruzmark, Alexandra K Mathis, Jay Herbst

**Affiliations:** 1 Department of Dermatology, HCA Florida Orange Park Hospital, Orange Park, USA; 2 Department of Dermatology, University of Illinois College of Medicine, Chicago, USA; 3 Department of Medicine, HCA Orange Park Florida, Orange Park, USA; 4 Dr. Philip Frost Department of Dermatology and Cutaneous Surgery, University of Miami Miller School of Medicine, Miami, USA

**Keywords:** dermatology, health disparities, mobile clinic, mohs surgery, rural medicine

## Abstract

Access to dermatologic care remains a persistent challenge in underserved communities, where workforce shortages, geographic barriers, and delayed referrals contribute to unmet medical and surgical needs. Mobile dermatology clinics represent a promising care-delivery model that brings specialty services directly to patients in these settings. Rather than replicating traditional clinic infrastructure, this editorial highlights the conceptual role of mobile dermatology clinics in improving access, reducing structural barriers, and supporting equitable delivery of dermatologic care. As health systems seek innovative strategies to address disparities, mobile clinics may serve as a flexible and scalable approach to expanding dermatologic services for underserved populations.

## Editorial

Access to dermatologic care, especially in underserved regions, remains a challenge, with a lack of available specialists and patients facing lengthy wait times and travel distances [1]. One study analyzing 811 mobile clinics across the United States reported a median number of 3491 visits annually with this healthcare delivery model. Fifty-nine percent of these visits served racial/ethnic minorities, and 41% of clients were uninsured [2], demonstrating the substantial reach of mobile clinics, their focus on underserved populations, and their potential to promote health equity and scalable healthcare delivery models. Notably, the recent World Health Assembly resolution recognized skin disease as a global health priority, emphasizing the need for innovative service delivery models for hard-to-reach populations [3]. Mobile dermatology clinics (MDCs) - converted recreational vehicles (RVs) designed to function as fully equipped dermatology offices - offer a solution.

An informal landscape assessment was completed, which revealed a very limited number of MDCs operating nationwide. OnSpot Dermatology is, to our knowledge, the only company that routinely delivers comprehensive dermatologic care through MDCs. Operating throughout Florida, OnSpot provides a full spectrum of services, including skin cancer screenings, lesion removals, biopsies, and select cosmetic procedures. Care is delivered from a renovated recreational vehicle outfitted as a fully functional dermatology clinic, and the practice accepts a wide range of insurance plans [4]. These MDCs represent an innovative approach to delivering care directly to patients and improving access to routine dermatologic services, including Mohs micrographic surgery (MMS).

**Figure 1:**
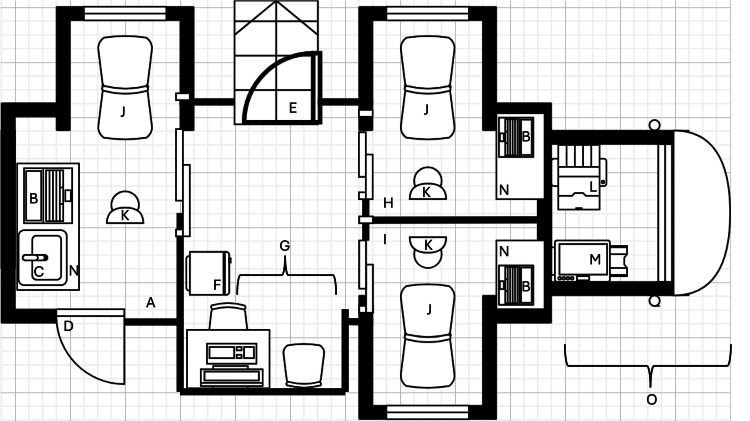
Schematic diagram of mobile dermatology clinic Schematic diagram of mobile dermatology clinic, adapted from OnSpot Dermatology [4]. A: Exam Room 1; B: Workstation; C: Sink; D: Elevator Lift; E: Entry; F: Medical Fridge; G: Intake Area; H: Exam Room 2; I: Exam Room 3; J: Exam Table; K: Chair; L: Printer; M: Copier; N: Table; O: Front of truck

Mobile dermatology clinics can contain multiple examination rooms capable of hosting routine dermatology visits or minor surgical procedures (Figure 1; the figure is provided solely to demonstrate the feasibility and potential scope of mobile dermatology clinic design, reflects an adapted schematic, and should not be interpreted as endorsement of any specific commercial entity or clinic model). For MMS, a separate mobile laboratory is parked alongside the main unit for histological analysis. MDCs mirror traditional dermatology practices (TDPs) with surgical tools and sterilization systems. MDCs are typically staffed with medical assistants, clerks to manage patient check-ins, Mohs histology technicians, and dermatologists. Patient flow is optimized through local business partnerships. Clinics are scheduled to visit central locations, where patients can conveniently access services, with vehicles operating in parking spaces. Offering MDCs at workplaces allows employees to receive dermatological care without leaving work, and medical institutions can extend their services to remote areas.

A significant advantage of MDCs is their opportunity to reduce barriers to care. In underserved regions, patients often face logistical challenges, such as long travel distances, to access dermatologic services [1]. MDCs can be equipped to facilitate minor excisions to complex Mohs procedures. This capability is particularly important in rural areas, where access to specialized surgical care is limited [1]. This could also be an addition to Street Medicine teams, which provide direct care to people experiencing homelessness, particularly since dermatology services are a largely unmet need [5].

Beyond improving geographic access, MDCs may promote earlier diagnosis and intervention, discouraging disease progression and morbidity. Underserved populations often present with more advanced skin disease due to limited specialty access [6]. By facilitating timely evaluations and follow-up care, MDCs may reduce disease severity at presentation and lessen the need for more extensive or costly interventions; however, this potential benefit is inferred conceptually from outcomes observed in teledermatology models that deliver care in local settings, rather than from direct evidence specific to MDCs [7]. Delivering care within the community may also improve patient trust and engagement, especially among individuals who have historically faced barriers or negative experiences within traditional healthcare settings [8]. Additionally, MDCs offer important public health and systems-level benefits. Mobile deployment represents an opportunity for clinics to target high-need areas and community centers, increasing the reach of preventative care and screening initiatives. This model may reduce downstream healthcare utilization related to untreated dermatologic disease, also supported by teledermatology models [9]. Notably, a Medicare analysis of the 2020-2023 Medicare Physician/Supplier Procedure Summary data demonstrated a 60-fold increase in procedures performed via mobile clinics across all provider types. The majority of procedural care was delivered by non-physicians [10], underscoring a critical opportunity for dermatologists to expand and lead the delivery of care within MDCs.

Mobile dermatology clinics require careful planning and investment. Converting and sustaining RVs into fully operational medical units involves significant funding sources. A retrospective cost assessment of mobile health clinics found that start-up expenses are primarily driven by the acquisition and outfitting of RV units, while ongoing operational costs are largely attributable to labor [11]. As normal laws and regulations in TDPs are applicable to MDCs, many of the costs applicable to opening a TDP must be considered, such as medical equipment, insurance, and consumables [12]. There are, however, some key differences between TDPs and MDCs. TDPs frequently rent office spaces, whereas MDCs are in RVs that must be purchased and may require permits. From a policy and reimbursement perspective, MDCs may face challenges related to payer recognition and billing logistics, areas in which data remains limited. Despite this, MDCs have shown great potential for scalability. The operational model can be expanded to include other medical specialties, and the flexibility of MDCs also positions them as aids in public health emergencies. 

Mobile dermatology clinics may offer an innovative and practical solution to address healthcare disparities by providing high-quality dermatologic care and surgical services directly to patients. While outcomes from MDCs have not been reported, mobile health clinics in other specialties have increased the number of screenings and enhanced chronic disease management, showing promise in enhancing clinical outcomes with the use of MDCs [13]. However, it is important to note that MDCs are an under-evaluated care model, necessitating research in their implementation and health services evaluation. Additionally, pre-emptive outreach for vulnerable populations has been shown to reduce downstream costs to the healthcare system [14]. By meeting patients where they are, MDCs demonstrate a forward-thinking approach to healthcare that holds significant promise for the future. To maximize their impact, successful MDC implementation requires careful consideration of regional healthcare infrastructure, resource availability, community engagement, and tailored operational strategies, underscoring the need for greater study and support of this care model across diverse settings.
